# Multi‐Center, Real‐World Registry Study of the UroLift Prostatic Urethral Lift (PUL) for Benign Prostatic Hyperplasia (BPH) in Japan

**DOI:** 10.1111/iju.70529

**Published:** 2026-06-15

**Authors:** Naoya Masumori, Satoru Takahashi, Go Anan, Fumiyasu Endo, Akira Furuta, Kazunori Haga, Mina Hatanaka, Shinobu Kato, Yoshihiro Komai, Yuki Kyoda, Nayuka Matsuyama, Shinichiro Murayama, Hirotaka Nagamatsu, Daisuke Obinata, Zenkichi Sekiguchi, Yuma Waseda

**Affiliations:** ^1^ Department of Urology Sapporo Medical University Sapporo Hokkaido Japan; ^2^ Department of Urology Nihon University School of Medicine Itabashi‐ku Tokyo Japan; ^3^ Department of Urology Yotsuya Medical Cube Chiyoda‐ku Tokyo Japan; ^4^ Department of Urology St. Luke's International Hospital Chuo‐ku Tokyo Japan; ^5^ Department of Urology Jikei University School of Medicine Minato‐ku Tokyo Japan; ^6^ Department of Urology Sanjukai Urological Hospital Sapporo‐shi Hokkaido Japan; ^7^ Urology, Saitama Sekishinkai Hospital Sayama‐shi Japan; ^8^ Kato Urological Clinic Hiratsuka‐shi Kanagawa Japan; ^9^ Komai Nephrology and Urology Clinic Osaka‐shi Osaka Japan; ^10^ Department of Urology Inuyama Chuo General Hospital Inuyama‐shi Aichi Japan; ^11^ Urology Department Tokyo International Ohori Hospital Mitaka‐shi Tokyo Japan; ^12^ Oita Urology Hospital Oita‐shi Oita Japan; ^13^ Department of Urology St. Marianna University Yokohama Seibu Hospital Yokohama‐shi Kanagawa Japan; ^14^ Department of Urology Institute of Science Tokyo Bunkyo‐ku Tokyo Japan

**Keywords:** benign prostatic hyperplasia, Japan, lower urinary tract symptoms, minimally invasive surgery, real world clinical trials

## Abstract

**Objectives:**

The prostatic urethral lift (PUL) for benign prostatic hyperplasia (BPH) has been shown to deliver rapid, durable symptom relief with low morbidity. Most studies have been performed in Western populations. The objective was to understand how PUL performs in the real world in Japan among a broad patient population.

**Methods:**

A post‐market registry study of consecutive PUL subjects across 14 Japanese centers was conducted. International Prostate Symptom Score (IPSS), quality of life (QOL), maximum flow rate (*Q*_max_) and sexual function were evaluated at baseline, 3‐ and 12‐ months post‐procedure. Paired *t*‐tests compared baseline and follow‐up data. Subject demographics, adverse events, BPH medication use, catheterization, and surgical retreatment were reported.

**Results:**

210 subjects were included. Baseline characteristics included age 74.3 ± 8.5 years, IPSS 18.0 ± 7.4, QOL 4.8 ± 1.3, *Q*_max_ 10.3 ± 5.5 mL/s and prostate volume 40.5 ± 16.1 cc. Paired analyses indicated IPSS improved 6.6 (36.9%), *p* < 0.0001; QOL improved 2.1 (43.3%), *p* < 0.0001 and *Q*_max_ improved 1.5 (14.7%) mL/s (*p* = 0.023) at 12 months. Sexual function measures were unchanged or significantly improved, although results should be interpreted with caution due to a high degree of missing data. BPH medication use decreased from 76.7% to 12.4%; One subject was surgically retreated by 12 months. Adverse events were typically mild–moderate and transient.

**Conclusions:**

This registry study from Japan indicates PUL is safe and effective; results corroborate those of previous studies. The data support the use of PUL in the broader BPH population in Japan.

**Trial Registration:**

Japan Registry of Clinical Trials (jRCT 2032220377)

## 1 Introduction

The prostatic urethral lift (PUL) for the treatment of lower urinary tract symptoms (LUTS) secondary to benign prostatic hyperplasia (BPH) has been available in Japan since April 2022. It has been proven to deliver rapid, significant and durable improvement with minimal morbidity and preservation of sexual function [1, 2, 3, 4, 5, 6]. While the evidence for PUL is extensive, most studies have been conducted in North America, Australia and Europe; less is known about outcomes in Japan where the investigations have mostly been small, single‐center studies [7, 8, 9]. Further, appropriate use criteria (AUC) for PUL in Japan are currently targeted toward BPH patients who are difficult to treat with conventional surgery (e.g., transurethral resection of the prostate (TURP) or holmium enucleation of the prostate (HoLEP)) for reasons such as significant comorbidities, on anticoagulation and/or advanced age. To better understand how PUL performs in the real world and its applicability to the broader BPH population in Japan, a multi‐center registry study was conducted after the device was cleared for use.

## 2 Methods

A post‐market registry study of consecutive PUL procedures performed across 14 centers in Japan beginning in June 2022 was conducted (jRCT 2 032 220 377). Subjects were enrolled both prospectively and retrospectively; those patients treated between the product launch date and facility contract date were enrolled retrospectively. All subjects included were diagnosed with LUTS secondary to BPH and provided informed consent. Charts at each site were reviewed after IRB approval, and the subjects were included in the registry. All surgeons were new PUL users and trained in the same manner prior to enrolling subjects.

**FIGURE 1:**
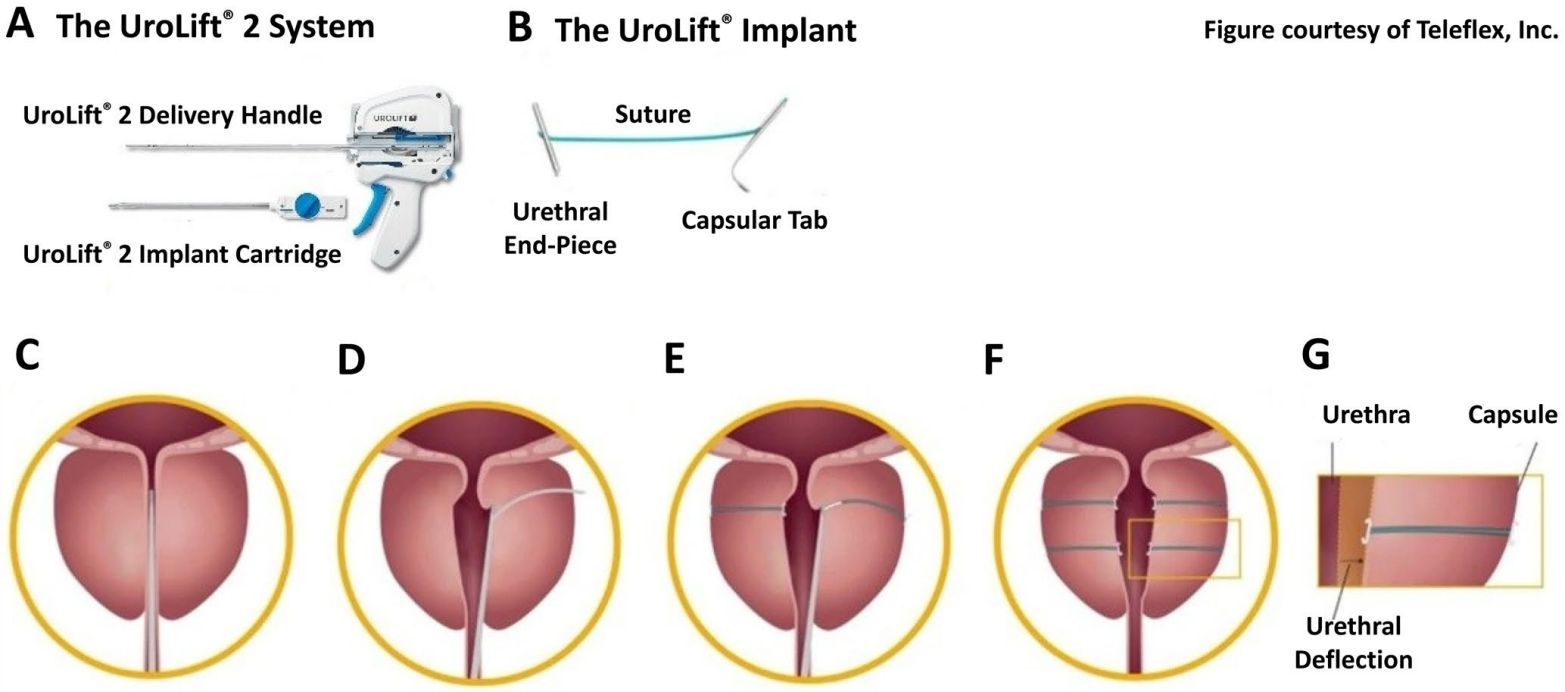
The UroLift 2 system and PUL procedure.

For this study, the UroLift2 System (Teleflex Interventional Urology, Pleasanton, California, USA) comprised of the UroLift 2 Delivery Handle and the UroLift 2 Implant Cartridge (which comes pre‐loaded with one UroLift Implant) (Figure 1A,B), was used. During the PUL procedure, under cystoscopic guidance, the delivery device is introduced through the sheath and angled to compress the prostate lobe (Figure 1C,D). Through the needle of the delivery device, the implant is deployed transprostatically and the process repeated until the required number of implants have been placed (Figure 1E,F). The implant attaches to the fibromuscular capsule on one end (with the capsular tab) and the more compliant periurethral tissue on the other end (with the urethral end‐piece), resulting in the opening of the prostatic urethra (Figure 1G).

Baseline subject demographics, prostate volume, prostate hypertrophy characteristics, uroflowmetry measures, PSA, medical history, urinary symptoms and severity scores, catheterization status and medication use data were collected. In cases of high PSA, pre‐operative prostate cancer assessment (e.g., digital rectal exam, MRI, etc.) was performed according to the investigator's standard of care and not based on a predefined protocol. Procedural details, hospital length of stay (LOS), post‐operative medication use, catheterization status, uroflowmetry and adverse events (AEs) were recorded at 3‐ and 12‐months post‐procedure. In this study, BPH medication use post‐procedure was physician‐directed based on real‐world clinical practice and not protocol‐driven. Retreatment through 12 months was also assessed by recording any instances of surgical treatment due to persistent or return of BPH/LUTS.

Urinary symptoms and severity were assessed using the International Prostate Symptoms Score (IPSS), Quality of Life Score (QOL) and the Overactive Bladder Symptoms Score (OABSS). Sexual function was evaluated using the International Index of Erectile Function short form (IIEF‐5) and the Male Sexual Health Questionnaire for ejaculatory function (MSHQ‐EjD). For the MSHQ‐EjD, the validated Japanese translation version was not available and a Japanese‐translated MSHQ‐EjD was provided. Urinary function was assessed using maximum urinary flow rate (Qmax) and post‐void residual volume (PVR). Baseline and follow up data were compared using two‐sided paired *t*‐tests where an a priori level of significance was set to 5%.

## 3 Results

A total of 210 subjects were included in the analysis, all of whom were Asian (see Table 1 for baseline characteristics). At the time of this report, baseline data for only 188 subjects had been submitted by the sites accounting for a baseline missingness of 22 (10.5%) subjects across all parameters; other data were missing for reasons including subjects not being assessed for a given parameter, and the details are shown in Table S1. The mean age at the time of procedure was 74.3 ± 8.5 years and the mean baseline pre‐procedure IPSS and QOL scores were 18.0 ± 7.4 and 4.8 ± 1.3, respectively. Qmax was 10.3 ± 5.5 mL/s, and PVR was 79.4 ± 99.2 mL. Average prostate volume was 40.5 ± 16.1 cc and subjects had varying prostate hypertrophy characteristics including lateral lobe hyperplasia (87.6%), intravesical protrusion of the prostate, IPP (28.6%), median lobe hypertrophy (13.3%), high bladder neck (12.4%) and obstructive median lobe, OML (1.0%). The assessment methods, IPP grading, overlap among prostate morphology categories and subgroup analyses comparing IPP or median lobe cases versus none are detailed in Supplementary Section A. Among the cohort, 7.6% were catheter‐dependent, 5.7% had LUTS‐related disturbed sleep and 2.9% reported related pelvic pain.

**TABLE 1:** Baseline characteristics.

Characteristic	*N* = 210
Age (years)	
Mean (SD) [Min, Max]	74.3 (8.5) [46.0, 92.0]
BMI	
Mean (SD) [Min, Max]	23.3 (2.9) [12.4, 32.2]
Missing subjects (%)	22 (10.5%)
Race	Number of Subjects (%)
Asian	188 (89.5%)
White	0 (0%)
Black	0 (0%)
Other	0 (0%)
Missing subjects (%)	22 (10.5%)
Prostate volume (cc)	
Mean (SD) [Min, Max]	40.5 (16.1) [12.0, 108.0]
Missing subjects (%)	23 (11.0%)
PSA (ng/mL)	
Mean (SD) [Min, Max]	3.5 (4.7) [0, 37.2]
Missing subjects (%)	43 (20.5%)
Qmax (mL/s)	
Mean (SD) [Min, Max]	10.3 (5.5) [0, 30.0]
Missing subjects (%)	45 (21.4%)
PVR (mL)	
Mean (SD)	79.4 (99.2) [0,585.0]
Missing subjects (%)	38 (18.1%)
IPSS total score	
Mean (SD) [Min, Max]	18.0 (7.4) [1,35]
Missing subjects (%)	44 (21.0%)
QOL total score	
Mean (SD) [Min, Max]	4.8 (1.3) [0,6]
Missing subjects (%)	44 (21.0%)
OABSS total score	
Mean (SD) [Min, Max]	6.7 (3.2) [1,14]
Missing subjects (%)	110 (52.4%)
IIEF total score	
Mean (SD) [Min, Max]	10.8 (7.5) [1,25]
Missing subjects (%)	116 (55.2%)
MSHQ‐EjD function	
Mean (SD) [Min, Max]	6.6 (4.4) [1,15]
Missing subjects (%)	172 (81.9%)
MSHQ‐EjD bother	
Mean (SD) [Min, Max]	1.8 (1.5) [0,5]
Missing subjects (%)	173 (82.4%)
Comorbidities number of subjects (%)	
Hypertension	70 (33.3%)
Diabetes mellitus	37 (17.6%)
Cancer	20 (9.5%)
Cerebrovascular disease	18 (8.6%)
Missing subjects (%)	22 (10.5%)
Urological conditions number of subjects (%)	
OAB	75 (35.7%)
Prostatitis	7 (3.3%)
Urethral stricture	6 (2.9%)
Prostate cancer	4 (1.9%)
Bladder stones	3 (1.4%)
Missing subjects (%)	22 (10.5%)
Urological history number of subjects (%)	
Prior urological surgery	27 (12.9%)
Prior radiation therapy	3 (1.4%)
Missing subjects (%)	24 (11.4%)
ASA physical status classification	
ASA I	48 (22.9%)
ASA II	95 (45.2%)
ASA III	28 (13.3%)
Missing subjects (%)	39 (18.6%)

At baseline, 76.7% of subjects were on medication for BPH of which the most common was alpha‐blocker (66.7%) followed by 5‐alpha reductase inhibitor (24.8%) and phosphodiesterase 5 inhibitor, PDE5 (20.5%); 15.7% of subjects were on medication for OAB. Also at baseline, 47 out of 210 subjects (22.4%) were on anti‐platelet and/or anticoagulation therapy. At the time of surgery (i.e., did not discontinue medications), 61.0% were on BPH medication and 7.6% were on OAB medication; 7.1% were on anti‐platelet therapy and 2.4% were anticoagulated.

PUL was conducted under general anesthesia in most cases (see Table 2). Overall mean procedure time was 51.3 ± 14.2 min, and an average of 4.1 ± 1.5 implants were placed. 68.1% of subjects were catheterized post‐procedure for an average of 1.36 ± 1.1 days (range 0–8), including subjects catheterized as standard of care which is typical in Japan. All 16 (7.6%) subjects who were on catheter pre‐procedure were catheterized post‐procedure for an average of 2.25 ± 2.0 days (range 1–8). The remaining 127 (60.5%) subjects were catheterized for an average of 1.24 ± 0.91 days (range 0–7). The mean hospital stay was 2.2 ± 2.2 days which is shorter than other more invasive BPH procedures in Japan.

**TABLE 2:** Procedure and peri‐procedural parameters.

Procedure information	*N* = 210
Type of anesthesia	
General	136 (64.8%)
Lumbar	28 (13.3%)
Local	8 (3.8%)
Periprostate nerve block	6 (2.9%)
Other	14 (6.7%)
Missing subjects (%)	23 (11.0%)
Overall procedure time (min)	
Mean (SD) [Min, Max]	51.3 (14.2) [15.0, 99.0]
Missing subjects (%)	23 (11%)
Time from delivery device insertion to final removal (min)	
Mean (SD) [Min, Max]	13.0 (7.8) [1.0, 50.0]
Missing subjects (%)	23 (11%)
Total number of implants	
Mean (SD) [Min, Max]	4.1 (1.5) [2, 8]
Missing subjects (%)	23 (11.0%)
Post‐procedure catheterization	
Subjects (%)	143 (68.1%)
Missing subjects (%)	24 (11.4%)
Length of stay (days)	
Mean (SD) [Min, Max]	2.2 (2.22) [0, 18]
Missing	23 (11.0%)

Paired analyses indicated significant improvement from baseline for all symptom measures at 3 and 12 months (Table 3). Mean IPSS improved by 7.1 (40.2%) and 6.6 (36.9%), *p* < 0.0001 for both; mean QOL improved by 2.2 (46.7%) and 2.1 (43.3%), *p* < 0.0001 for both and mean OABSS improved by 1.2 (16.3%) and 1.2 (17.8%), *p* = 0.001 for both, at 3 and 12 months, respectively. Flowmetry measures were also significantly improved from baseline; Qmax improved by 2.5 (23.6%), *p* < 0.0001 and 1.5 (14.7%) mL/s (*p* = 0.023) and PVR decreased by 35.6 (44.3%) and 39.0 (46.7%) mL, *p* < 0.0001 for both, at 3 and 12 months, respectively (Table 3). MSHQ‐EjD function significantly improved by 2.3 (31.9%), *p* = 0.009 and 3.5 (58.5%), *p* = 0.003 points at 3 and 12 months, respectively; while the improvement in MSHQ‐EjD bother was not great enough to be deemed significant at 3 months (−0.4, −28.2%, *p* = 0.110), the improvement was significant by 12 months (−1.0, −63.6%, *p* = 0.010). There was no significant change in IIEF from baseline at either time point.

**TABLE 3:** Comparison of Outcomes from Baseline to 3 and 12 Months Follow Up.

Measure	3 Months	12 Months
IPSS		
Subjects (paired)	149	105
Mean baseline (SD)	17.8 (7.3)	18.0 (7.2)
Mean follow up (SD)	10.6 (6.6)	11.3 (6.6)
Mean change (paired)	−7.1	−6.6
Mean % change (paired)	−40.2%	−36.9%
*p*‐value for mean change (vs. baseline)	*p* < 0.0001	*p* < 0.0001
QOL		
Subjects (paired)	149	105
Mean baseline (SD)	4.7 (1.3)	4.8 (1.3)
Mean follow up (SD)	2.5 (1.6)	2.7 (1.7)
Mean change	−2.2	−2.1
% change	−46.7%	−43.3%
*p*‐value for mean change (vs. baseline)	*p* < 0.0001	*p* < 0.0001
OABSS		
Subjects (paired)	56	38
Mean baseline (SD)	7.4 (3.1)	6.7 (2.7)
Mean follow up (SD)	6.2 (3.3)	5.5 (3.0)
Mean change	−1.2	−1.2
% change	−16.3%	−17.8%
*p*‐value for mean change (vs. baseline)	*p* = 0.001	*p* = 0.002
*Q*_max_, mL/s		
Subjects (paired)	149	101
Mean baseline (SD)	10.6 (5.4)	10.2 (5.3)
Mean follow up (SD)	13.1 (6.6)	11.7 (5.3)
Mean change	+2.5	+1.5
% change	+23.6%	+14.7%
*p*‐value for mean change (vs. baseline)	*p* < 0.0001	*p* = 0.023
PVR (mL)		
Subjects (paired)	160	111
Mean baseline (SD)	80.3 (101.6)	83.5 (94.9)
Mean follow up (SD)	44.7 (73.0)	44.5 (62.4)
Mean change	−35.6	−39.0
% change	−44.3%	−46.7%
*p*‐value for mean change (vs. baseline)	*p* < 0.0001	*p* < 0.0001

The percentage of subjects taking BPH medications and OAB medications decreased over time (Table 4). One subject (1/210 or 0.5%) was surgically retreated with HoLEP for bladder outlet obstruction.

**TABLE 4:** Change in medication use and surgical retreatment through 12 months.

Measure (% subjects)	Baseline	3 Mos	12 Mos
BPH medication use	76.7%	13.8%	12.4%
Alpha blocker	66.7%	11.0%	10.0%
5ARI	24.8%	1.9%	2.9%
PDE5 inhibitor	20.5%	3.3%	2.9%
Other	1.9%	1.0%	1.0%
Missing data	11.0%	12.4%	37.6%
OAB medication use	15.7%	12.9%	10.0%
Beta 3 adrenoceptor agonist	11.4%	12.4%	9.0%
Anticholinergic	4.8%	1.4%	1.4%
Other drug	1.0%	0.5%	0.5%
Missing data	11.0%	12.4%	37.6%
Surgical retreatment	NA	Not recorded	0.5%
Missing data	NA	Not recorded	18.1%

13 (6.2%) subjects were re‐catheterized within 3 months post‐procedure; 12 were re‐catheterized for 11.4 ± 16.1 (range 1–60) days. 1 subject was prescribed self‐catheterization as needed at home but, due to his dementia, an indwelling catheter was later placed, and no further follow up data were available.

Two related serious adverse events (SAE) occurred in 2 subjects (Table 5). One subject experienced pelvic hematoma requiring hospitalization; the subject recovered without further issues. One subject experienced clot urinary retention; he was hospitalized for 7 days during which time spontaneous voiding was restored after 5 days of catheterization. 3 subjects had unrelated SAE (cerebral infarction, acute pyelonephritis and death from a traffic accident).

**TABLE 5:** Adverse events (AEs).

	0–3 Months		4–12 Months	
	Events	Subjects (% of 210 total)	Events	Subjects (% of 210 total)
Serious AEs	3	3 (1.4%)	2	2 (1.0%)
Related AEs	2	2 (1.0%)	0	0 (0.0%)
All AEs	30	29 (13.8%)	9	8 (3.8%)
Related AEs	19	19 (9.5%)	1	1 (0.5%)
Urinary retention (incl clot urinary retention)		11 (5.2%)		0 (0.0%)
OAB (frequency, urgency)		4 (1.9%)		0 (0.0%)
Hematuria		2 (1.0%)		0 (0.0%)
Pelvic hematoma		1 (0.5%)		0 (0.0%)
Pelvic pain syndrome		1 (0.5%)		0 (0.0%)
Pelvic pain		0 (0.0%)		1 (0.5%)

Less serious AEs (e.g., urinary retention, OAB symptoms) were typically mild to moderate and resolved within 2 weeks. There was no incidence of confirmed, de novo sustained ejaculatory or erectile dysfunction.

8 (3.8%) subjects experienced bleeding‐related AEs (e.g., hematuria, pelvic hematoma, clot urinary retention). These subjects were older (mean 79, range 68–91 years) with mean prostate volume 39 cc (range 20 cc‐70 cc) including 2 subjects with prostate volume ≥ 50 cc (59 cc and 70 cc). 4 subjects were taking BPH medication and 3 were taking OAB medication prior to PUL treatment.

47 (22.4%) subjects were on antiplatelet and/or anticoagulation therapy at baseline of which 5 experienced bleeding‐related AEs (1–7 days post‐procedure). 17 out of 47 subjects did not discontinue antiplatelet and/or anticoagulation therapy during surgery and 3 out of these 17 subjects experienced bleeding‐related AEs. Among those 3 subjects, 1 had pelvic hematoma (SAE discussed previously) 3 days post‐procedure and recovered after 4 days of hospitalization; 1 experienced clot urinary retention 1‐day post‐procedure and recovered after 1 day of catheterization. The third subject had hematuria 1‐day post‐PUL when he tried to remove the post‐procedure catheter that was placed routinely and recovered after 2 more days of catheterization; hematuria was not present previously and this event was not related to the procedure.

There were 6 instances (in 6 subjects) of UroLift System device malfunction that all occurred during the procedure. In 2 instances, there was an inability to expose the capsular tab to the outside of the prostate and in 2 instances, an inability to expose the capsular tab from the needle of the delivery device. In one case, there was a loose urethral end‐piece and in another case, an inability to move the trigger. No AEs occurred as a result of device malfunction.

## 4 Discussion

Our study findings are consistent with controlled and real‐world studies performed in Western populations [1, 2, 3, 4, 5, 6] that indicate PUL significantly improves symptom and flowmetry measures through 12 months. While sexual function was preserved in subjects with data, these results are exploratory and limited due to the high degree of missing data. Safety and efficacy were demonstrated across a broad range of demographics and suggests that BPH patients outside the current AUC parameters may benefit from PUL treatment with the UroLift System. Notably, this study included a broad age range (46–92 years), subjects with larger prostates up to 108.0 cc, those who were catheterized and who were on anticoagulation/antiplatelet therapy.

Surgical retreatment was also low (1/210 or 0.5%) but these results must be interpreted with caution due to the significant data loss; if we consider only the 172 subjects with reintervention data at 12 months, the retreatment rate may be restated as 0.6% (1/172). The percentage of subjects on BPH medications decreased from 76.7% to 12.4% by 12 months. Whether these subjects on BPH medications by 12 months needed them or never discontinued from pre‐procedure is unknown.

In current practice, some Japanese surgeons select patients with prostate volume < 50 cc as candidates for PUL. To explore the effects of prostate volume, we performed an ad hoc analysis comparing subjects with prostate volume < 50 cc (*n* = 147) and prostate volume ≥ 50 cc (*n* = 40). We found no significant difference in IPSS (−7.1 ± 7.1 vs. −7.4 ± 8.5, *p* = 0.81), QOL (−2.2 ± 1.8 vs. −2.4 ± 1.8, *p* = 0.59), Qmax (2.5 ± 7.1 vs. 2.6 ± 4.9 mL/s, *p* = 0.93) or PVR (−29.0 ± 94.9 vs. −65.2 ± 110.5 mL, *p* = 0.07) change from baseline to 3 months or from baseline to 12 months (IPSS −6.6 ± 7.1 vs. −6.7 ± 7.7, *p* = 0.98) (QOL −2.0 ± 1.9 vs. −2.3 ± 2.1, *p* = 0.39) (Qmax 1.5 ± 7.0 vs. 1.5 ± 4.3 mL/s, *p* = 0.96) (PVR −28.5 ± 94.8 vs. −71.1 ± 111.9 mL, *p* = 0.06) between prostates < 50 cc and prostates ≥ 50 cc, respectively. Among subjects not in retention pre‐procedure, 67% with prostate volume < 50 cc were catheterized compared to 70% with prostate volume ≥ 50 cc (*p* = 0.75); there was also no significant difference in duration of post‐procedure catheterization between groups (1.2 ± 0.93 vs. 1.3 ± 0.84 days, *p* = 0.97). There was no significant difference in the proportion of subjects with related AEs among subjects with prostates < 50 cc and those with prostates ≥ 50 cc (10.9% vs. 12.5%, *p* = 0.78, respectively). Among the 2 subjects with SAEs, one had prostate volume < 50 cc (pelvic hematoma thought to be due to the delivery device being pressed too hard against the prostate adenoma during implant deployment); the other had prostate volume ≥ 50 cc (clot urinary retention believed to be related to bleeding from the needle puncture site). For the 8 subjects with bleeding‐associated related AEs/SAEs (hematuria, pelvic hematoma, clot urinary retention), 6 had prostate volume < 50 cc while the remaining 2 had prostate volume ≥ 50 cc. Given this post hoc analysis and the small sample size of subjects with prostate volume ≥ 50 cc, the results are limited but seem to corroborate a previous real‐world, multi‐center PUL study in Japan that found no significant difference in urinary status improvement (IPSS, QOL, Qmax) at 1 month and 3 months across groups with prostate volumes < 30, 30 to < 50 and ≥ 50 mL [10]. Still, any conclusions regarding subgroup findings should be interpreted cautiously in light of their exploratory nature.

The limitations of this study include incomplete baseline data collection and incomplete data or loss to follow up at 12 months which reflects the nature of a real‐world study. Missing data could bias the findings and was present within the registry despite all reasonable efforts being made to limit their occurrence. To determine whether missing data affect the results, effectiveness outcomes at 12 months were compared using a modified “intent to treat” analysis in which the last available data is carried forward for subjects with baseline data and a modified “per protocol” analysis in which only patients with baseline and 12‐month data were analyzed. The results showed no significant difference (Table S2). In addition, a comparison of baseline characteristics between completers and non‐completers indicated no significant difference between completers and non‐completers (Table S3).

Also, since the validated Japanese translation version of the MSHQ‐EjD was not available, a Japanese‐translated version was used; since this version is non‐validated, results must be interpreted with caution and further study is warranted. In addition, medication usage is not always reliably reported in electronic medical records so the data must be interpreted with caution. Another potential concern is the effect of the PUL implants on MRI imaging or radical prostatectomy in the event of future prostate cancer. Since published studies have demonstrated the implants cause artifact only in the posterior transition zone where the incidence of a single focus of prostate cancer is very low (and targeted biopsy could be performed if needed) [11] and that the implants can be removed without interference in the event of surgery [1], we believe these concerns are limited. The strengths of this study are the large number of subjects and centers included and that the results reflect outcomes in a heterogeneous population in real clinical practice.

In conclusion, PUL in an unconstrained real‐world population seems to be safe and effective, and we believe a broad range of BPH patients in Japan could benefit from PUL (including patients with prostates ≥ 50 cc). Patients on antiplatelet/anticoagulation therapy may also be treated, although special care should be taken with this patient population. Findings regarding preservation of sexual function should be interpreted with caution due to the high degree of missing data.

## Author Contributions

**Go Anan:** investigation. **Mina Hatanaka:** investigation. **Fumiyasu Endo:** investigation. **Kazunori Haga:** investigation. **Shinichiro Murayama:** investigation. **Naoya Masumori:** conceptualization, supervision, writing – review and editing. **Satoru Takahashi:** conceptualization, supervision, writing – review and editing. **Akira Furuta:** investigation. **Yuki Kyoda:** investigation. **Yuma Waseda:** investigation. **Shinobu Kato:** investigation. **Zenkichi Sekiguchi:** investigation. **Daisuke Obinata:** investigation. **Yoshihiro Komai:** investigation. **Hirotaka Nagamatsu:** investigation. **Nayuka Matsuyama:** investigation.

## Funding

This study was funded by Teleflex Medical Japan.

## Disclosure

Naoya Masumori is the Editor‐in‐Chief of the International Journal of Urology and first author of this article. He was excluded from editorial decision‐making related to the acceptance and publication of this article. Satoru Takahashi and Daisuke Obinata are the Editorial Board members of the International Journal of Urology and another first author and the co‐authors of this article, respectively. To minimize bias, they were excluded from all editorial decision‐making related to the acceptance of this article for publication.

## Ethics Statement

All subjects provided informed consent. Approval was granted by the central IRB. (No. MINS‐EC‐230216).

## Conflicts of Interest

Author N.M. has received lecture fees from Teleflex Medical Japan, Kissei Pharmaceutical Co. Ltd. and Astellas Pharma Inc.; author S.T. holds leadership and advisory positions with Kissei Pharmaceutical Co. Ltd. and Astellas Pharma Inc.; author G.A. has received lecture fees from Teleflex Medical Japan, Kissei Pharmaceutical Com. Ltd. and Kyorin Pharmaceutical Co. Ltd.; author K.H. has received lecture fees from Boston Scientific Corporation; author D.O. has received lecture fees from Teleflex Medical Japan, Tsumura & Co., Eisai Co. Ltd., Boston Scientific Corporation and Kyorin Pharmaceutical Co. Ltd. All other authors declare no conflicts of interest.

## Supporting information


**Table S1:** Reasons for missing data.
**Table S2:** Sensitivity analysis comparing modified intent to treat with modified per protocol.
**Table S3:** Baseline Characteristics for completers and non‐completers.

## Data Availability

Research data are not shared.
