# Supplementary material for: Multi‐Center, Real‐World Registry Study of the UroLift Prostatic Urethral Lift (PUL) for Benign Prostatic Hyperplasia (BPH) in Japan
Source: Int J Urol. 2026 Jun 15;33(6):e70529. doi: 10.1111/iju.70529 (PMC13267450; doi:10.1111/iju.70529)
Supplement: Supplementary file 1 — Table S1: Reasons for missing data. Table S2: Sensitivity analysis comparing modified intent to treat with modified per protocol. Table S3: Baseline Characteristics for completers and non‐completers. [file IJU-33-0-s001.docx]

**Supplementary Table 1: Reasons for Missing Data**

|  | | | **IPSS** |  | **QOL** |  | **Qmax** |  | **PVR** |  | **MSHQ-EjD Function** |  | **MSHQ-EjD Bother** |  | **IIEF** |  |
| --- | --- | --- | --- | --- | --- | --- | --- | --- | --- | --- | --- | --- | --- | --- | --- | --- |
|  |  |  | Baseline (N) | 12 Months | Baseline (N) | 12 Months | Baseline | 12 Months | Baseline | 12 Months | Baseline | 12 Months | Baseline | 12 Months | Baseline | 12 Months |
| **Subjects with data** | | | **166** | **114** | **166** | **115** | **165** | **112** | **172** | **120** | **38** | **30** | **37** | **30** | **94** | **48** |
| **Subjects missing data** | | | **44** | **96** | **44** | **95** | **45** | **98** | **38** | **90** | **172** | **180** | **173** | **180** | **116** | **162** |
| **Total subjects** | | | **210** | **210** | **210** | **210** | **210** | **210** | **210** | **210** | **210** | **210** | **210** | **210** | **210** | **210** |
| *Breakdown of missing data* | | |  |  |  |  |  |  |  |  |  |  |  |  |  |  |
|  | Site has not entered data | | 23 (52.3%) | 58 (60.4%) | 23 (52.3%) | 58 (61.1%) | 22 (48.9%) | 58 (59.2%) | 22 (57.9%) | 58 (64.4%) | 23 (13.4%) | 58 (32.2%) | 23 (13.3%) | 58 (32.2%) | 23 (19.8%) | 58 (35.8%) |
|  | Test not performed | | 21 (47.7%) | 12 (12.5%) | 21 (47.7%) | 11 (11.6%) | 23 (51.1) | 15 (15.3%) | 16 (42.1%) | 8 (8.9%) | 149 (86.6%) | 75 (41.7%) | 150 (86.7%) | 75 (41.7%) | 93 (80.2%) | 77 (47.5%) |
|  | Subject missed visit but continued | | 0 | 3 (3.1%) | 0 | 3 (3.2%) | 0 | 3 (3.1%) | 0 | 3 (3.3%) | 0 | 3 (1.7%) | 0 | 3 (1.7%) | 0 | 3 (1.9%) |
|  | Subject missed visit and withdrew | | 0 | 16 (16.7%) | 0 | 16 (16.8%) | 0 | 15 (15.3%) | 0 | 15 (16.7%) | 0 | 18 (10.0%) | 0 | 18 (10.0%) | 0 | 17 (10.5%) |
|  | Subject doing well, no follow up required | | 0 | 2 (2.1%) | 0 | 2 (2.1%) | 0 | 2 (2.0%) | 0 | 1 (1.1%) | 0 | 3 (1.7%) | 0 | 3 (1.7%) | 0 | 2 (1.2%) |
|  | Moved or transferred | | 0 | 3 (3.1%) | 0 | 3 (3.2%) | 0 | 3 (3.1%) | 0 | 3 (3.3%) | 0 | 3 (1.7%) | 0 | 3 (1.7%) | 0 | 3 (1.9%) |
|  | Death |  | 0 | 1 (1.0%) | 0 | 1 (1.1%) | 0 | 1 (1.0%) | 0 | 1 (1.1%) | 0 | 1 (0.6%) | 0 | 1 (0.6%) | 0 | 1 (0.6%) |
|  | Doctor moved hospitals, could not find subject | | 0 | 1 | 0 | 1 (1.1%) | 0 | 1 (1.0%) | 0 | 1 (1.1%) | 0 | 1 (0.6%) | 0 | 1 (0.6%) | 0 | 1 (0.6%) |
|  | MSHQ: No sexual activity past month | | 0 | 0 | 0 | 0 | 0 | 0 | 0 | 0 | 0 | 18 (10.0%) | 0 | 18 (10.0%) | 0 | 0 |
|  | **Total subjects missing data** | | **44** | **96** | **44** | **95** | **45** | **98** | **38** | **90** | **172** | **180** | **173** | **180** | **116** | **162** |

**Supplementary Table 2: Sensitivity Analysis Comparing Modified Intent to Treat with Modified Per Protocol**

|  | **Modified Intent to Treat (mITT)** | --> | Use subjects with baseline data, 3 month F/U and 12 month F/U | |  |
| --- | --- | --- | --- | --- | --- |
|  |  |  | Carry forward the last available F/U data | |  |
|  |  |  | (If only 3 mo F/U is available, use it for 12 mo F/U) | |  |
|  |  |  |  | |  |
|  | **Modified Per Protocol (mPP)** | --> | Use subjects with baseline data and 12 month F/U | |  |
|  |  |  |  |  | |
|  | **IPSS** | **QOL** | **Qmax** | **PVR** | |
| mITT: 12 months - baseline | N=154, Mean=-6.7, SD=7.4 | N=154, Mean=-2.0, SD=1.9 | N=156, Mean=1.4, SD=6.1 | N=167, Mean=-32.6, SD=94.6 | |
|  |  |  |  |  | |
| mPP: 12 months - baseline | N=115, Mean=-6.5, SD=7.0 | N=115, Mean=-2.0, SD=1.9 | N=110, Mean=1.3, SD=6.3 | N=120, Mean=-37.7, SD=97.8 | |
|  |  |  |  |  | |
| p-value comparison mITT vs mPP | 0.823 | 1 | 0.897 | 0.657 | |

**Supplementary Table 3: Baseline Characteristics for Completers and Non-Completers**

|  | **IPSS** | | **QOL** | | **OABSS** | | **Qmax** | | **PVR** | |
| --- | --- | --- | --- | --- | --- | --- | --- | --- | --- | --- |
|  | With 12 Mo Data | No 12 Mo Data | With 12 Mo Data | No 12 Mo Data | With 12 Mo Data | No 12 Mo Data | With 12 Mo Data | No 12 Mo Data | With 12 Mo Data | No 12 Mo Data |
| Subjects with baseline data (N) | 105 | 61 | 105 | 61 | 38 | 62 | 101 | 64 | 111 | 61 |
| Age | N=105, 73.17±7.79 | N=61, 72.92±8.97 | N=105,  73.17±7.79 | N=61,  72.92±8.97 | N=38,  73.66±8.24 | N=62,  73.66±6.91 | N=101,  73.39±7.84 | N=64,  73.69±9.03 | N=111,  73.46±7.93 | N=61,  74.20±8.83 |
|  | P=0.849 | | P=0.849 | | P=0.998 | | P=0.821 | | P=0.576 | |
| BMI | N=105, 23.69± 2.89 | N=61,  23.08 ± 2.84 | N=105,  23.69±2.89 | N=61,  23.08±2.84 | N=38,  23.22±2.67 | N=62,  23.50±2.91 | N=101,  23.65±2.90 | N=64,  22.85±2.66 | N=111,  23.51±2.99 | N=61,  22.85±2.51 |
|  | P=0.190 | | P=0.190 | | P=0.632 | | P=0.078 | | P=0.125 | |
| Prostate volume (cc) | N=105, 41.19±15.98 | N=61,  38.41±15.26 | N=105,  41.19±15.98 | N=61,  38.41±15.26 | N=38,  43.26±16.88 | N=62,  43.79±17.35 | N=101,  41.22±15.33 | N=64,  37.17±14.50 | N=111,  40.99±15.90 | N=61,  36.77±12.55 |
|  | P=0.273 | | P=0.273 | | P=0.894 | | P=0.094 | | P=0.075 | |
| PSA (ng/mL) | N=102,  3.43±4.28 | N=48,  3.27±3.44 | N=102,  3.43±4.28 | N=48,  3.27±3.44 | N=36,  3.39±4.25 | N=50,  3.70±4.51 | N=99,  3.37±4.17 | N=53,  2.95±3.43 | N=108,  3.17±3.98 | N=50,  3.14±3.63 |
|  | P=0.826 | | P=0.826 | | P=0.747 | | P=0.533 | | P=0.961 | |
| Qmax (mL/s) | N=102, 10.26±5.29 | N=54,  11.09±5.73 | N=102,  10.26±5.29 | N=54,  11.09±5.73 | N=36,  9.28±3.88 | N=57,  10.50±5.41 | N=101,  10.20±5.26 | N=64,  10.48±5.84 | N=108,  9.87±4.91 | N=57,  11.15±6.38 |
|  | P=0.362 | | P=0.362 | | P=0.245 | | P=0.751 | | P=0.188 | |
| PVR (mL) | N=102, 75.67±75.54 | N=57,  63.96±96.15 | N=102,  75.67±75.54 | N=57  63.96±96.15 | N=36,  93.67±84.74 | N=59,  79.17±91.15 | N=101,  79.25±82.85 | N=64,  77.27±119.46 | N=111, 83.45±94.90 | N=61,  72.03±106.92 |
|  | P=0.398 | | P=0.398 | | P=0.442 | | P=0.907 | | P=0.472 | |
| IPSS | N=105, 17.96±7.21 | N=61,  17.95±7.77 | N=105,  17.96±7.21 | N=61,  17.95±7.77 | N=38,  19.84±6.76 | N=62,  18.97±7.08 | N=98,  18.24±7.05 | N=58,  16.91±7.88 | N=104,  18.56±7.15 | N=55,  16.29±7.66 |
|  | P=0.993 | | P=0.993 | | P=0.543 | | P=0.277 | | P=0.065 | |
| QOL | N=105,  4.75±1.28 | N=61,  4.79±1.21 | N=105,  4.75±1.28 | N=61,  4.79±1.21 | N=38,  4.92±1.26 | N=62,  5.00±1.13 | N=98,  4.80±1.28 | N=58,  4.64±1.25 | N=104,  4.83±1.24 | N=55,  4.56±1.30 |
|  | P=0.865 | | P=0.865 | | P=0.746 | | P=0.453 | | P=0.213 | |

**Supplementary Section A: Prostate Morphology Details and Comparison of Outcomes**

Assessment methods: IPP, median lobe hypertrophy, obstructive median lobe and high bladder neck were determined by each investigator's judgment based on pre-procedural imaging (cystoscopy, TRUS, transabdominal ultrasound, CT and/or MRI).

Grading: IPP was measured in mm and graded as follows: I (<5mm), n=12; II (5-10mm), n=38; III (>10mm), n=10

Overlap: The overlap among groups was as follows: Median lobe hypertrophy (n=28), Median lobe and IPP (n=22), Median lobe and obstructive median lobe (n=2), Median lobe, OML, IPP (n=1)

Subgroup analyses: The subgroup analyses for subjects with median lobe hypertrophy vs none and for IPP vs none are shown in the tables below. In both cases, there was no significant difference in outcomes between groups.

There was one subject retreated with HoLEP for bladder outlet obstruction. This subject had lateral lobe hypertrophy, median lobe hypertrophy and IPP grade II (10mm) but did not have obstructive median lobe. The occurrence of retreatment through 12 months was too low to perform a subgroup analysis.

**Comparison of Outcomes for Subjects with Median Lobe Hypertrophy vs No Median Lobe Hypertrophy**

|  |  | **Median Lobe Hypertrophy** |  |  | **No Median Lobe Hypertrophy** |  |  |  |
| --- | --- | --- | --- | --- | --- | --- | --- | --- |
| **IPSS Total Score** | **N** | **Mean Change** | **Std Dev** | **N** | **Mean Change** | **Std Dev** |  | **P-value for difference in means** |
| 3 Months - Baseline | 24 | -6.13 | 5.74 | 125 | -7.34 | 7.62 |  | 0.458 |
| 12 Months - Baseline | 21 | -4.85 | 6.13 | 85 | -7.04 | 7.39 |  | 0.223 |
| **QOL** |  |  |  |  |  |  |  |  |
| 3 Months - Baseline | 24 | -2.13 | 1.33 | 125 | -2.23 | 1.9 |  | 0.740 |
| 12 Months - Baseline | 20 | -1.85 | 1.81 | 85 | -2.11 | 2.0 |  | 0.603 |
| **Qmax, mL/s** |  |  |  |  |  |  |  |  |
| 3 Months - Baseline | 24 | 3.09 | 5.84 | 125 | 2.38 | 6.89 |  | 0.638 |
| 12 Months - Baseline | 21 | 0.64 | 5.26 | 80 | 1.69 | 6.68 |  | 0.506 |
| **PVR, mL** |  |  |  |  |  |  |  |  |
| 3 Months - Baseline | 26 | -47.96 | 121.58 | 134 | -33.19 | 93.79 |  | 0.486 |
| 12 Months - Baseline | 25 | -54.08 | 117.59 | 87 | -34.71 | 95.39 |  | 0.405 |

**Comparison of Outcomes for Subjects with IPP vs no IPP**

|  |  | **IPP** |  |  | **No IPP** |  |  | |
| --- | --- | --- | --- | --- | --- | --- | --- | --- |
| **IPSS Total Score** | **N** | **Mean Change** | **Std Dev** | **N** | **Mean Change** | **Std Dev** |  | **P-value for difference in means** |
| 3 Months - Baseline | 47 | -7.21 | 7.06 | 102 | -7.12 | 7.51 |  | 0.942 |
| 12 Months - Baseline | 41 | -6.51 | 7.23 | 64 | -6.69 | 7.24 |  | 0.904 |
|  |  |  |  |  |  |  |  |  |
| **QOL** |  |  |  |  |  |  |  |  |
| 3 Months - Baseline | 47 | -2.28 | 2.02 | 102 | -2.19 | 1.73 |  | 0.779 |
| 12 Months - Baseline | 41 | -1.95 | 2.17 | 64 | -2.13 | 1.84 |  | 0.661 |
|  |  |  |  |  |  |  |  |  |
| **Qmax** |  |  |  |  |  |  |  |  |
| 3 Months - Baseline | 46 | 2.82 | 4.48 | 103 | 2.36 | 7.52 |  | 0.642 |
| 12 Months - Baseline | 39 | 1.58 | 4.35 | 62 | 1.4 | 7.45 |  | 0.877 |
|  |  |  |  |  |  |  |  |  |
| **PVR** |  |  |  |  |  |  |  |  |
| 3 Months - Baseline | 48 | -47.96 | 132.03 | 112 | -30.29 | 80.22 |  | 0.3 |
| 12 Months - Baseline | 43 | -50.28 | 126.94 | 68 | -31.71 | 79.35 |  | 0.394 |
